# Phenotypic Spectrum, Diagnostic Challenges, and Clinical Outcomes in Stiff Person Syndrome: A Single-Center Case Series

**DOI:** 10.3390/neurolint18080143

**Published:** 2026-07-28

**Authors:** M-Isabel Eraso, Angela Carolina-Rosero, Alma-Fuentes, Melissa-Luque, Maria Angelica-Coronel, Luis Fontanilla, Juan Camilo Rodriguez, Narledys Bravo Nunez

**Affiliations:** 1 Facultad de Ciencias de la Salud, Universidad Simón Bolívar, Barranquilla 080001, Colombia; angela.rosero@unisimon.edu.co (A.C.-R.); alma.fuentes@unisimon.edu.co (A.-F.); melissa.luque@unisimon.edu.co (M.-L.); maria.coronel1@unisimon.edu.co (M.A.-C.); luis.fontanilla@unisimon.edu.co (L.F.); juan.rodriguezc@unisimon.edu.co (J.C.R.); narledis.nunez@unisimon.edu.co (N.B.N.); 2 Clínica la Misericordia Internacional, Barranquilla 080001, Colombia

**Keywords:** stiff-person syndrome, anti-GAD antibodies, autoimmune neurological disorders, muscle rigidity, GABA

## Abstract

**Introduction:** Stiff-Person Syndrome (SPS) is a rare autoimmune neurological disorder characterized by progressive muscle rigidity and painful spasms, primarily affecting axial and proximal musculature. Its diagnosis can be challenging due to clinical overlap with other neurological conditions such as spasticity, dystonia, or functional movement disorders. The detection of antibodies against glutamic acid decarboxylase (anti-GAD) is a key biomarker that supports diagnosis. **Methods:** Two clinical cases of patients with manifestations consistent with classic SPS are described, and were evaluated in a specialized neurology service. Both patients underwent detailed clinical assessment, complementary studies, and serum testing for anti-GAD antibodies. **Results:** Both patients presented with progressive rigidity and fluctuating muscle spasms, predominantly involving axial musculature. After an extensive diagnostic workup, elevated anti-GAD antibody titers were documented in both cases, confirming the diagnosis of classic SPS. Treatment with medications enhancing GABAergic neurotransmission was associated with significant clinical improvement, evidenced by reduced rigidity and the decreased frequency of spasms. **Conclusions:** These cases highlight the importance of considering SPS in the differential diagnosis of progressive rigidity syndromes. Identification of anti-GAD antibodies is essential for diagnostic confirmation, and treatment that aims to enhance GABAergic neurotransmission can significantly improve symptoms and patient functionality.

## 1. Introduction

Stiff-Person Syndrome (SPS) is a rare autoimmune neurological disorder characterized by progressive muscle rigidity, episodic painful spasms, and continuous motor unit activity on electromyography [1]. SPS has been recognized as a disorder within the spectrum of central motor inhibition, primarily associated with dysfunction of inhibitory neurotransmission mediated by gamma-aminobutyric acid (GABA) [2]. Clinically, SPS manifests with persistent co-contraction of agonist and antagonist muscles, leading to lumbar hyperlordosis, gait impairment, and heightened sensitivity to external stimuli. Diagnosis is primarily based on the criteria proposed by Dalakas, which include: (I) stiffness involving axial and limb muscles, particularly the thoracolumbar and abdominal regions; (II) painful spasms triggered by unexpected sensory or emotional stimuli; (III) continuous motor unit activity demonstrated by electromyography; (IV) absence of alternative neurological disorders explaining the clinical presentation; (V) positive serological markers, most commonly anti-GAD65 or anti-amphiphysin antibodies; and (VI) clinical improvement following benzodiazepine therapy. The condition is most commonly associated with autoantibodies against glutamic acid decarboxylase 65 (GAD65), although antibodies targeting amphiphysin, glycine receptor, and other synaptic proteins have also been described [3].

Because of its low prevalence, clinical heterogeneity, and overlap with other neurological disorders—including spasticity, dystonia, functional movement disorders, and myelopathies—the diagnosis of SPS is often significantly delayed. This diagnostic delay may contribute to progressive motor impairment, increased disability, and substantial deterioration in patients’ quality of life [4].

In this context, early recognition of clinical manifestations and identification of immunological biomarkers are essential for establishing the diagnosis and guiding timely treatment.

- **Case 1**

A 32-year-old Caucasian male was evaluated in an outpatient setting with a history of epilepsy and hypothyroidism under treatment, presenting with a 5-year history of progressive clinical symptoms consisting of rigidity in the left lower limb, limiting initiation and maintenance of gait, associated with paroxysmal episodes of sustained, painful, and involuntary muscle contractions. The condition progressed to involve bilateral lower limb rigidity, with involuntary muscle contractions extending from the lumbar region to the lower extremities, triggered by auditory, tactile, thermal stimuli, and stressful situations.

**Figure 1:**
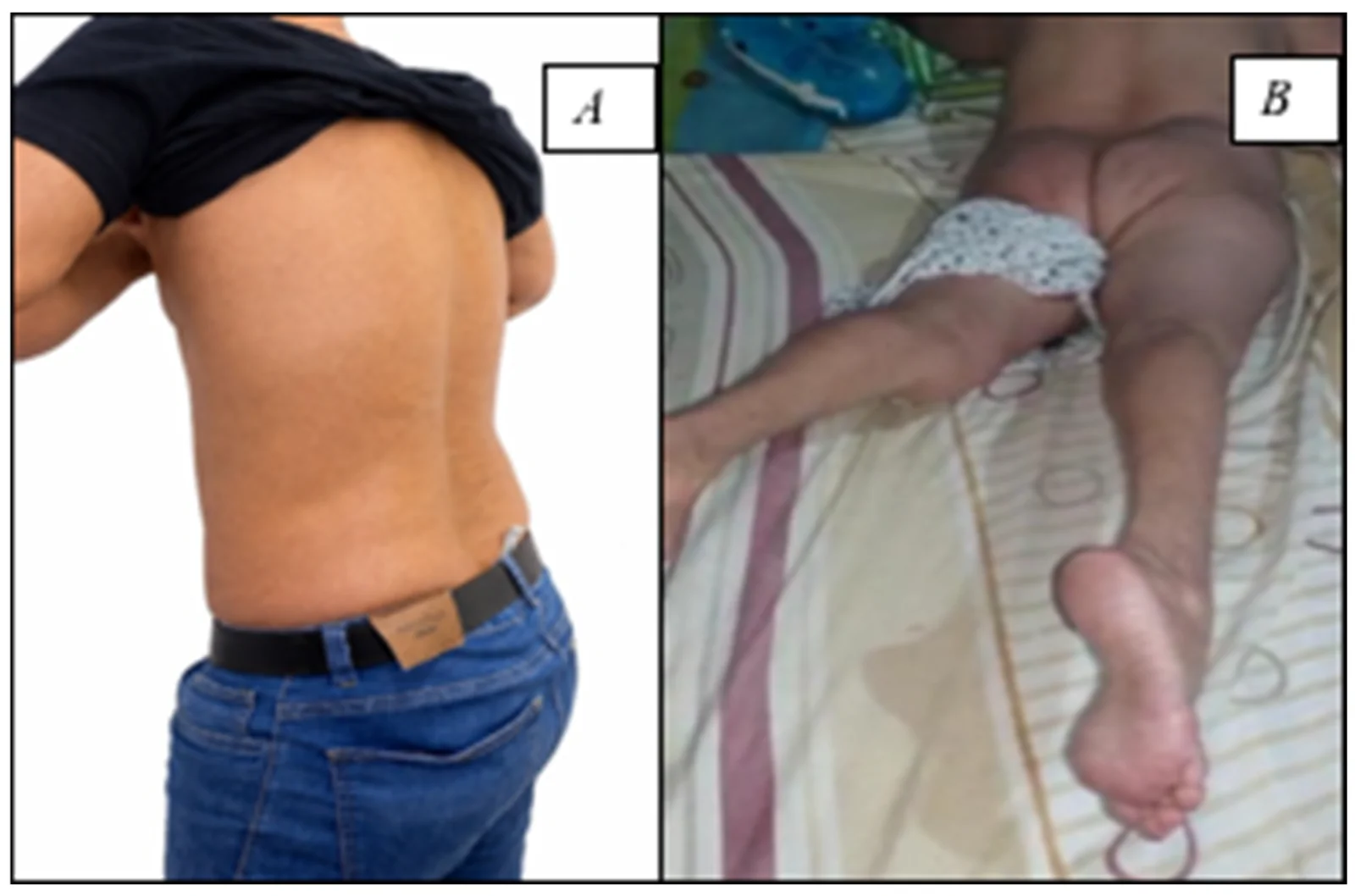
Comparative image of lumbar hyperlordosis due to sustained contraction of the lumbar musculature. (**A**) Case 1. (**B**) Case 2.

**Figure 2:**
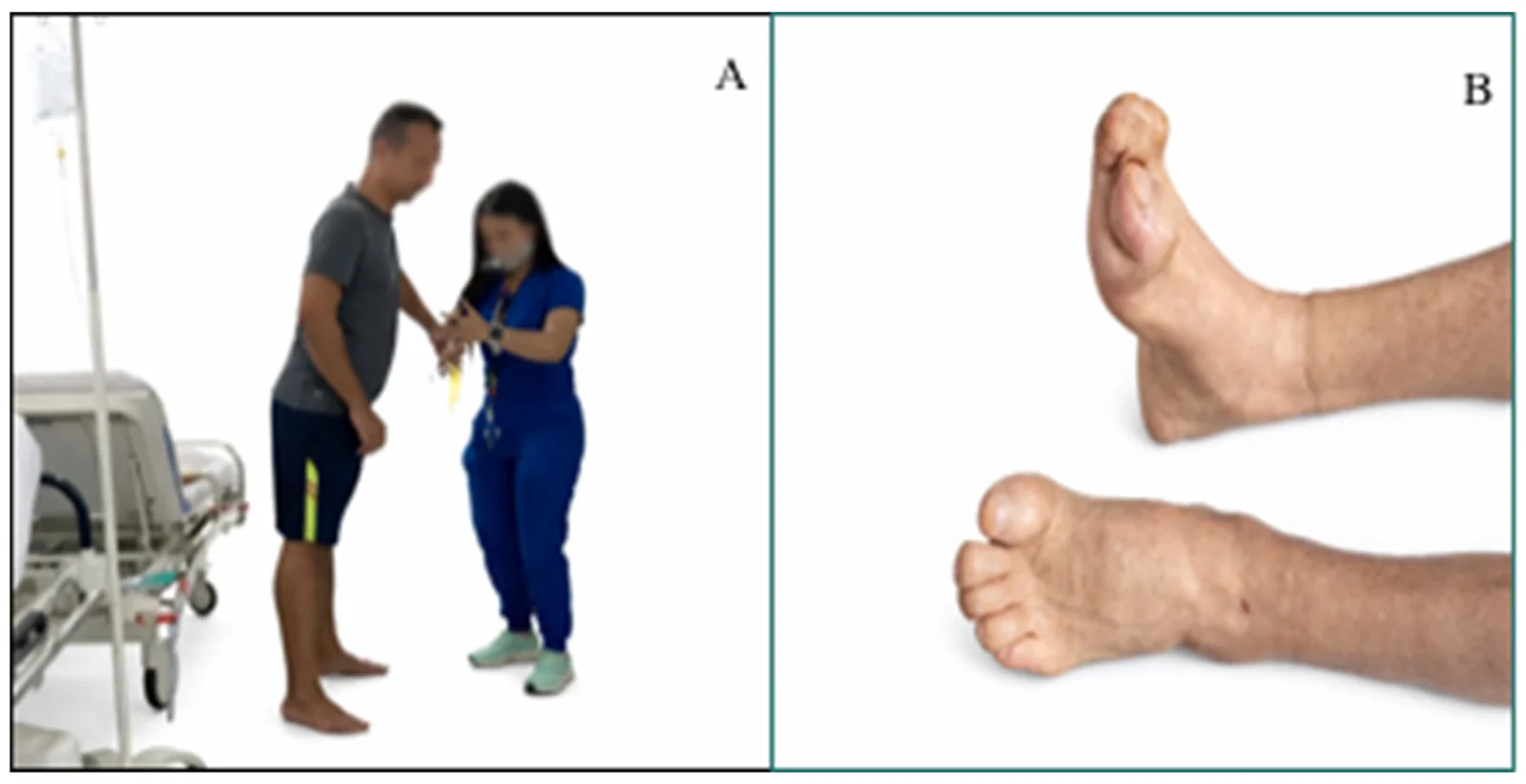
(**A**) Case 1: Rigidity due to contraction of the agonist and antagonist muscles of the lower extremities leading to postural instability. (**B**) Case 2: Foot deformity in both feet.

The patient reported frequent falls, dysautonomic symptoms including diaphoresis, and mood changes described as emotional lability, depressed affect, and excessive worry. On neurological examination, increased muscle tone was observed along with hypertrophy of the lumbar paravertebral muscles and hyperlordosis, associated with reduced range of motion. Gait assessment revealed a widened base of support, en bloc movement, and impaired turning (Figure 1A and Figure 2A).

Paraclinical studies, including thyroid profile, vitamin B12, B1 immunoassay, and total CPK automated enzymatic method, were within normal limits. Serum testing for anti-amphiphysin antibodies was negative, while GAD antibodies (GAD-Abs) were positive at titers of 93.04 U/mL. Neurophysiological studies demonstrated spontaneous activity characterized by large, polyphasic motor unit potentials, without electrical silence at rest. During voluntary activation, increased amplitude and polyphasic motor units were identified. Neuroimaging studies, including brain and whole neuroaxis MRI, showed no structural abnormalities.

Pharmacological therapy was initiated with a GABA agonist (clonazepam 1 mg orally every 8 h), and clinical improvement was observed within 24 h of treatment initiation.

- **Case 2**

A 69-year-old caucuses female was evaluated outpatient setting, presented with a 7-year history of progressive symptoms, initially characterized by increased muscle tone and distal-predominant mobility limitation in the right lower limb. Six months later, symptoms progressed to the contralateral lower limb, resulting in impaired gait maintenance and frequent falls. Additionally, she developed paroxysmal episodes of painful muscle contractions involving the abdominal, paravertebral, and lower limb muscles.

Approximately three years after symptom onset, the patient developed functional impairment affecting basic activities such as walking and bathing independently, with a Barthel Index score of 65. She received treatment with muscle relaxants (baclofen, 10 mg every 8 h) and GABAergic agents (gabapentin, 300 mg every 12 h), and lamotrigine, 100 mg every 12 h, was added during the last year due to suspected seizure activity. However, symptoms persisted, along with fear of leaving home related to her condition.

On physical examination, increased muscle tone was observed, associated with reduced muscle trophism in the lower limbs, lumbar hyperlordosis, and deformity of the plantar arch and metatarsals of the right foot (Figure 1B and Figure 2B). Paraclinical studies, including thyroid profile, vitamin B12, B1, immunoassay folic acid, and creatine kinase levels (automated enzymatic method), were within normal limits. Serum testing revealed positive GAD antibodies (GAD-Abs) at 98.81 U/mL, while anti-amphiphysin antibodies were negative.

Neurophysiological studies have demonstrated high-frequency polyphasic motor unit potentials in the absence of voluntary activity and without evidence of electrical silence. Myotonic discharges were identified in the right vastus lateralis, brachioradialis, and biceps muscles. During voluntary activation, polyphasic motor units with increased amplitude were observed. Neuroimaging studies, including brain and whole neuroaxis MRI, showed no structural lesions.

A therapeutic trial with the GABA agonist clonazepam, 0.5 mg orally every 12 h, combined with baclofen, 10 mg every 8 h, was initiated, resulting in a gradual improvement in lumbar pain and progressive overall symptom improvement.

Table 1 presents a summary of the Dalakas criteria used to establish the diagnosis of stiff-person syndrome (SPS) in cases 1 and 2.

## 2. Discussion

Stiff-Person Syndrome (SPS) refers to a rare (orphan) disease that has gained increasing recognition in recent years [4], although its diagnosis is still frequently overlooked due to limited awareness of this entity. Its main clinical features include rigidity of the limbs, axial and abdominal muscles and intense, painful spasms exacerbated by tactile, auditory or emotional stimuli, along with gait disturbances secondary to these core symptoms [5].

The etiology of SPS remains unknown; however, GAD antibodies (GAD-Abs)—directed against an enzyme that is rate-limiting in the synthesis of GABA, a key inhibitory neurotransmitter—play a fundamental role in its pathophysiology. These antibodies are also implicated in other conditions such as epilepsy, ataxia, and limbic encephalitis, as well as in endocrine disorders including type 1 diabetes mellitus, hypothyroidism, pernicious anemia, and vitiligo. These conditions may precede the onset of SPS symptoms by up to two years, as observed in our patient, who had a history of epilepsy and hypothyroidism [4,6].

SPS spectrum disorders can be classified according to the distribution of rigidity, the presence of associated neurological manifestations, the presence or absence of GAD-Abs and other antibodies, and the presence of underlying neoplasms [7,8]. The most commonly recognized phenotype is classic SPS, in which muscle spasms and rigidity predominantly involve axial and limb muscles, particularly the lower extremities. Continuous contraction of agonist and antagonist muscles underlies paraspinal muscle rigidity, which may lead to the characteristic lumbar hyperlordosis seen in this condition, as well as to muscle hypertrophy and gait disturbances [9,10].

SPS typically presents between the third and sixth decades of life and has an estimated prevalence of 1–2 cases per million [11], with women being more frequently affected than men at a ratio of 2:1 [12]. In adults, the median time from symptom onset to diagnosis is approximately 6.2 years [13].

The most commonly recognized phenotype is classic SPS, in which muscle spasms and rigidity predominantly involve axial and limb muscles, especially the lower extremities [10]. Continuous contraction of agonist and antagonist muscles explains the rigidity of paraspinal muscles, which can result in characteristic lumbar hyperlordosis, muscle hypertrophy, and subsequent gait impairment [9].

Although SPS is characterized by paroxysmal muscle contractions that are typically axial and symmetric, up to 30% of patients may exhibit asymmetric involvement of the hands and feet, with postures including foot dorsiflexion, extension, and slight abduction of the contralateral leg [14]. Cases of foot deformities have been reported in phenotypes such as SPS with cerebellar features and have been associated with delayed diagnosis and a more complex disease course with difficult functional recovery [12].

In addition to motor symptoms, it is important to recognize psychiatric manifestations, which occur in a large proportion of patients with SPS and significantly impact quality of life and prognosis. In a series of 239 patients with SPS, the prevalence of associated psychiatric conditions was reported as follows: anxiety in 56%, depression in 45%, and agoraphobia in 24% [15], Management of these symptoms is challenging, as there is evidence that serotonin and norepinephrine reuptake inhibitors—commonly used for anxiety and depression—may worsen SPS symptoms [11], making psychotherapy a fundamental component of treatment.

Dysautonomic symptoms during episodes of muscle contraction are not uncommon and may include hypertension, tachycardia, hyperthermia, and diaphoresis. In the case of our male patient, painful muscle spasms were accompanied by diaphoresis [16].

Table 2 outlines the clinical manifestations of stiff-person syndrome (SPS) observed in the present case report.

When establishing the diagnosis with Dalakas criteria, it is important to consider GAD-Abs titers, since, as previously mentioned, they may be present in other disorders; however, in SPS, anti-GAD levels are typically 50 times higher than average, whereas in type 1 diabetes mellitus and hypothyroidism they increase approximately 10-fold [7]. Radioimmunoassay is the most sensitive and specific method for detecting anti-GAD antibodies, with a sensitivity of 96% and a specificity of 95% [9].

The presence of anti-GAD antibodies is not required for the diagnosis of stiff-person spectrum disorder. Nevertheless, case–control cohort studies have reported that serum anti-GAD titers >1000 U/mL, as measured by ELISA, exhibit a sensitivity of 98% and specificity of approximately 50% for the classical phenotype [4].

Paraneoplastic variants are uncommon, accounting for less than 10% of all patients with SPS. Anti-amphiphysin antibodies are the most frequently identified in these cases. However, a small proportion—between 4% and 6%—of patients with GAD-Abs-positive SPS may have associated malignancies, including thymoma, breast, thyroid, kidney, and colon cancer [2].

In electrodiagnostic studies, the expected EMG findings in SPS include motor unit potential with normal configuration and firing rates without evidence of denervation. However, the persistence of motor unit potentials at rest, together with inappropriate activation of antagonist muscles due to failure of inhibitory reflex mechanisms, results in continuous motor activity characterized by high-frequency discharges known as “continuous motor unit activity” [17].

In some cases reported in the literature, EMG findings have shown intermittent biphasic contractions at rest without clear demonstration of co-contraction of agonist and antagonist muscles. Additionally, decreased recruitment of motor units and reflex hyperexcitability of spinal motor neurons have been observed. These findings may be related to dysfunction in interneuronal circuits, which are thought to use GABA as a neurotransmitter, particularly involving inhibitory spinal interneuronal circuits [18,19].

Pathological spontaneous activity such as fibrillation potentials, fasciculations, myokymia, and neuromyotonic or myotonic discharges is not a typical finding in SPS. However, cases have been reported in which conditions such as myotonic dystrophy and SPS coexist, possibly due to the shared autoimmune origin of both diseases [20].

Regarding treatment, given the rarity of stiff-person syndrome (SPS), there are currently no randomized controlled trials comparing different therapeutic strategies. Symptomatic management with benzodiazepines is considered the first-line option with favorable responses reported in 78–100% of patients. However, side effects such as somnolence often limit tolerability, so treatment is typically initiated at low doses with gradual titration [2]. In refractory cases of SPS, clonidine has demonstrated clinical benefit in several case reports. Neuropharmacological evidence suggests that α2-adrenergic stimulation may suppress spinal reflex activity and promote muscle relaxation, thereby contributing to symptomatic improvement [21].

Immunomodulatory therapies have been proposed for patients who fail to achieve adequate symptomatic control. However, initiation of these treatments should not be unduly delayed, given the progressive nature of SPS. Intravenous immunoglobulin (IVIg) remains the treatment supported by the highest level of evidence in SPS, whereas rituximab is increasingly used in refractory disease despite the absence of large randomized controlled trials. More recently, FcRn-targeting therapies such as efgartigimod have emerged as promising therapeutic alternatives, although evidence is currently limited to mechanistic studies and early clinical experience [13,22].

Management decisions are based primarily on individual case reports. To date, treatment is based on four main pillars: suppression of the autoimmune process through immunotherapy; symptomatic control of rigidity and spasms; management of mood disorders in conjunction with psychiatry when present; treatment of the underlying cause in paraneoplastic SPS; and, importantly, supportive rehabilitation [9].

Up to 35.2% of patients with stiff-person syndrome (SPS) may develop respiratory symptoms. Patients with involvement of more than five body regions have a significantly higher risk of acute crisis-related respiratory failure, which may progress to ventilatory failure. Therefore, early immunomodulatory therapy should be considered in patients at risk [23].

SPS is a chronic condition that significantly affects patients’ quality of life, impacting not only physical functioning, but also social functioning, often forcing patients to withdraw from social and professional activities. Psychiatric symptoms, which are highly prevalent in individuals with SPS, further reduce quality-of-life scores. This was demonstrated in a study of 24 SPS patients assessed using the Short Form Health Survey (SF-36), which confirmed this strong correlation [24].

In neurological practice, one or two cases may be encountered throughout an entire career [25]. In this report, two patients with SPS, presenting typical clinical features and positive GAD-Abs, were identified within a one-year period, making this description particularly noteworthy.

This underscores the importance of recognizing this rare syndrome, which affects a small proportion of the population but has devastating consequences for patients and their families. Early diagnosis and timely treatment improve prognosis and quality of life.

## 3. Conclusions

Stiff-Person Syndrome (SPS) remains an uncommon but potentially disabling autoimmune neurological disorder whose diagnosis is frequently delayed because of its clinical overlap with spasticity, dystonia, functional movement disorders, and other causes of progressive rigidity. The present cases underscore the importance of maintaining a high index of suspicion in patients presenting with progressive axial or lower-limb rigidity, stimulus-sensitive painful spasms, lumbar hyperlordosis, and gait impairment, particularly when routine neuroimaging and laboratory investigations are unrevealing. Recognition of these characteristic clinical features, together with early application of the Dalakas diagnostic criteria, may substantially shorten the diagnostic pathway.

Our findings further support the value of combining careful neurological examination with targeted neurophysiological and serological investigations. Continuous motor unit activity on electromyography and the detection of anti-GAD antibodies remain key diagnostic indicators that can facilitate early confirmation of SPS and reduce the risk of misdiagnosis. Importantly, the presence of psychiatric manifestations, including anxiety, depression, and maladaptive behavioral responses to chronic disability, should be systematically assessed, as these symptoms may represent an integral component of the disease spectrum and significantly affect quality of life.

From a therapeutic perspective, early initiation of GABA-enhancing agents, such as clonazepam and baclofen, may provide substantial symptomatic benefit and prevent progressive functional deterioration. Prompt treatment may also reduce the risk of secondary musculoskeletal complications, including fixed postural abnormalities and orthopedic deformities resulting from chronic muscle rigidity and recurrent spasms. Although both patients achieved satisfactory clinical control with symptomatic therapy alone, treatment strategies should be individualized according to disease severity, functional impairment, antibody profile, and response to first-line agents, with immunomodulatory therapies reserved for refractory or progressive cases.

Taken together, these cases highlight three practical principles for clinical practice: (i) early recognition of the characteristic triad of rigidity, painful stimulus-triggered spasms, and exaggerated startle responses; (ii) prompt confirmation through electromyographic and immunological testing; and (iii) timely initiation of targeted therapy aimed at restoring inhibitory GABAergic function. Adoption of this approach may facilitate earlier diagnosis, improve functional outcomes, and ultimately reduce long-term disability in patients with SPS.

## 4. Limitations

This study has several limitations. First, the small number of patients, inherent to the case series design, limits the generalizability of the findings. Second, the observational nature of the study precludes establishing causal relationships between therapeutic interventions and the observed clinical improvement. Finally, the lack of long-term follow-up restricts the evaluation of sustained therapeutic response and disease progression.

Despite these limitations, the presented cases highlight the importance of considering Stiff-Person Syndrome in the differential diagnosis of progressive rigidity syndromes and underscore the diagnostic value of anti-GAD antibodies, as well as the favorable response to treatments aimed at enhancing GABAergic neurotransmission.

## Figures and Tables

**Table 1:** Dalakas diagnostic criteria.

Dalakas	Case 1	Case 2
Rigidity in axial muscles, mainly involving the paraspinal, abdominal, and thoracolumbar muscles, leading to a fixed deformity (hyperlordosis)	+	+
Superimposed painful spasms triggered by unexpected noises, emotional stress, or tactile stimuli	+	+
Confirmation of continuous motor unit activity in agonist and antagonist muscles on electromyography	+	+
Confirmation of continuous motor unit activity in agonist and antagonist muscles on electromyography	Absent	Absent
Positive serology for GAD65 (or amphiphysin) autoantibodies, assessed by immunocytochemistry, Western blot, or radioimmunoassay	+ GAD 65	+ GAD 65
Clinical response to diazepam	+Clonazepam	+ Clonazepam

**Table 2:** Physical examination findings in patients with SPS.

Symptoms	Case 1	Case 2
Increased tone in axial/truncal muscle groups	+	+
Increased tone in the legs (symmetric or asymmetric)	+	+
Normal strength in upper and lower limbs (except in advanced stages of the disease)	+	Advanced stage
Possible hyperreflexia, without plantar extension	+	+
Normal sensory function and coordination	+	+
Lumbar spine hyperlordosis (resulting from contraction of abdominal and paraspinal muscles)	+	+
“Wooden” sensation on muscle palpation due to spasms	+	+
Slow, wide-based, and cautious gait	+	Barthel scale 65 points
Intact cognitive function	+	+
Normal sphincter function	+	+

## Data Availability

The datasets generated and/or analyzed during the current study are not publicly available due to patient privacy and ethical considerations. As this work involves clinical case data with a risk of participant identification, no additional data can be shared beyond those presented in the manuscript. Access to further information is restricted in accordance with institutional ethical requirements and the informed consent obtained from the participants.

## Abbreviations

The following abbreviations are used in this manuscript:

GABA	γ-aminobutyric acid
SPS	Stiff person spectrum
EMG	Electromyography
Anti-GAD	Glutamic acid decarboxylase antibodies
GAD-Abs	Glutamic acid decarboxylase antibodies
